# Comparison of the Tibial Posterior Slope Angle Between the Tibial Mechanical Axis and Various Diaphyseal Tibial Axes After Total Knee Arthroplasty

**DOI:** 10.1016/j.artd.2022.06.015

**Published:** 2022-09-19

**Authors:** Yoshinori Ishii, Hideo Noguchi, Junko Sato, Ikuko Takahashi, Hana Ishii, Ryo Ishii, Kei Ishii, Shin-ichi Toyabe

**Affiliations:** a Ishii Orthopaedic & Rehabilitation Clinic, Gyoda, Saitama, Japan; b Kanazawa Medical University, School of Plastic Surgery, Ishikawa, Japan; c Shinshu University Hospital, Nagano, Japan; d Iwate Prefectural Ninohe Hospital, Ninohe, Iwate, Japan; e Niigata University Crisis Management Office, Niigata University Hospital, Niigata University Graduate School of Medical and Dental Sciences, Niigata, Japan

**Keywords:** Total knee arthroplasty, Posterior tibial slope, mechanical axis, diaphyseal axis, full-length lateral tibial radiograph, lateral view of standard knee radiograph

## Abstract

**Background:**

The posterior tibial slope angle (PTS) is crucial for sagittal alignment after total knee arthroplasty (TKA). This study aimed to determine which PTS based on the lateral view of standard knee radiographs (LSKRs; 36 × 43 cm) reflects the PTS based on a full-length lateral tibial radiograph (FLTR).

**Methods:**

A total of 290 patients (355 knees) who underwent primary TKA were retrospectively recruited. Cross-sectional views from the 3-dimensional digital model of the tibial prosthesis and bone complex in the sagittal plane were used as FLTRs and LSKRs. Considering the region 21.5 cm proximal to the site of FLTR as the spot for LSKR to determine the 5 tibial diaphyseal axes, the axis that simulates the PTS as determined by the tibial mechanical axis between the center of the tibial component and the ankle plafond in LSKR was determined and compared.

**Results:**

PTS (α_5_) defined by the line connecting the midpoints of tibial width between the region 10-cm distal to the knee joint and the distal end of the tibia based on LSKR revealed the least mean difference (0.13° ± 1.00°) and the strongest correlation (*P* < .001, r = 0.948) with PTS based on FLTR (α_0_). The number of knees in α_5_, indicating a difference of <2° from α_0_, was 333 of 355 (93.8%). The equivalence test results indicated that α_0_ and α_5_ were statistically equivalent within a difference of 2° (*P* < .001).

**Conclusions:**

PTS (α_5_) can be used as a clinically reliable substitution of the true PTS on an FLTR for evaluating sagittal alignment after TKA.

## Introduction

The posterior tibial slope angle (PTS) is crucial for the sagittal alignment of the lower extremity. It is defined as the angle between the knee joint line and mechanical axis of the tibia. It is essential to ensure the proper sagittal alignment when performing total knee arthroplasty (TKA). PTS is a major factor in the long-term success of TKA because of its effects on kinematics and mechanics [[1], [2], [3]], moment arm [4], and sagittal gap imbalance [5]. The optimal angles range between <5° and ≥8° depending on the design concept of each TKA [1,2,4]. The full-length lateral tibial radiograph (FLTR) provides an accurate representation of the sagittal alignment because it is easy to determine the mechanical axis of the tibia between the center of the tibial component and ankle plafond after TKA.

FLTRs are usually not taken routinely to evaluate alignment after TKA [[6], [7], [8]]; the lateral view of standard knee radiographs (LSKRs; 36 × 43 cm) is commonly used instead. Because the long radiograph is often used in more difficult cases, LSKRs are frequently used in daily clinical practice during postoperative follow-up periods because of logistical and technical problems [7], convenience, and lower cost [8]. Even recent studies [9,10] have evaluated the PTS after TKA using LSKR. However, the PTS using LSKR should be regarded as the estimation of the true PTS using FLTR as LSKR without the ankle plafond cannot determine the mechanical axis of the tibia. Thus, to date, several studies have evaluated the correlations between FLTR and LSKR and shown different results in native knee [6], anterior cruciate ligament (ACL)-deficient knee [11,12], and osteoarthritic (OA) knee [13]. The tibial axis based on LSKR has been determined by connecting 2 points among the midpoints of the tibial width at sites 5, 10, or 15 cm distal from the joint line and the distal end of radiographs [[11], [12], [13]] or tibial cortex line [14,15] to obtain the ideal PTS with the least error compared with the true PTS based on FLTR. However, to our knowledge, a method to measure the PTS based on LSKR, which can substitute the true PTS based on FLTR, has not been established yet.

This study aimed to determine which PTS based on LSKR reflects the most approximate PTS using FLTR images obtained from a quantitative 3-dimensional (3D) assessment using computed tomography (CT) images after TKA.

## Material and methods

The relevant institutional review board approved this study (ID no. 2021-1). All patients signed a consent form that included a description of the protocol. A total of 290 patients (355 knees) who had undergone hybrid (cemented tibia, uncemented femur) primary TKA with the New Jersey LCS total knee system (DePuy, Warsaw, IN) between March 2011 and December 2021 were recruited for this retrospective, cross-sectional study. All patients had been preoperatively diagnosed with knee osteoarthritis on plain radiographic findings. The exclusion criteria were a history of knee or tibial/fibular surgery that may have affected the PTS, such as TKA, tibial osteotomy, and tibial/fibular fractures. The patients’ clinical characteristics are summarized in Table 1.

**Table 1:** Patient demographics.

Characteristics (*N* = 290 patients, 355 knees)	Mean (SD)
Age at the first TKA	74 (8)
Sex; male/female, patients (knees)	46 (55)/ 244 (300)
Body height (cm)	151 (7)
Body weight (kg)	61 (12)
BMI (kg/m^2^)	26 (4)

BMI, body mass index; SD, standard deviation.

All surgeries were performed by a single surgeon using a standardized technique with the standard medial parapatellar approach, including the necessary soft-tissue release for proper gap balancing with mechanical alignment principles under tourniquet control. Proximal tibial osteotomy was performed perpendicular to the mechanical axis of the limb with a 10° posterior slope in the sagittal plane using an extramedullary guide. With the knee in 90° flexion, the anteroposterior femoral cutting block was positioned relative to the anterior cortex of the femur using a femoral intramedullary alignment rod. The femoral positioner was used to ensure that the anterior and posterior femoral resections were parallel to the tibial resection. Distal femoral osteotomy was performed using a 6° valgus cutting guide. In all knees, the femoral components were fixed without cement, and the tibial components were fixed with cement. The patella was not resurfaced, and no lateral retinaculum release was performed in any case.

### Measurement and definition of PTS

**Figure 1:**
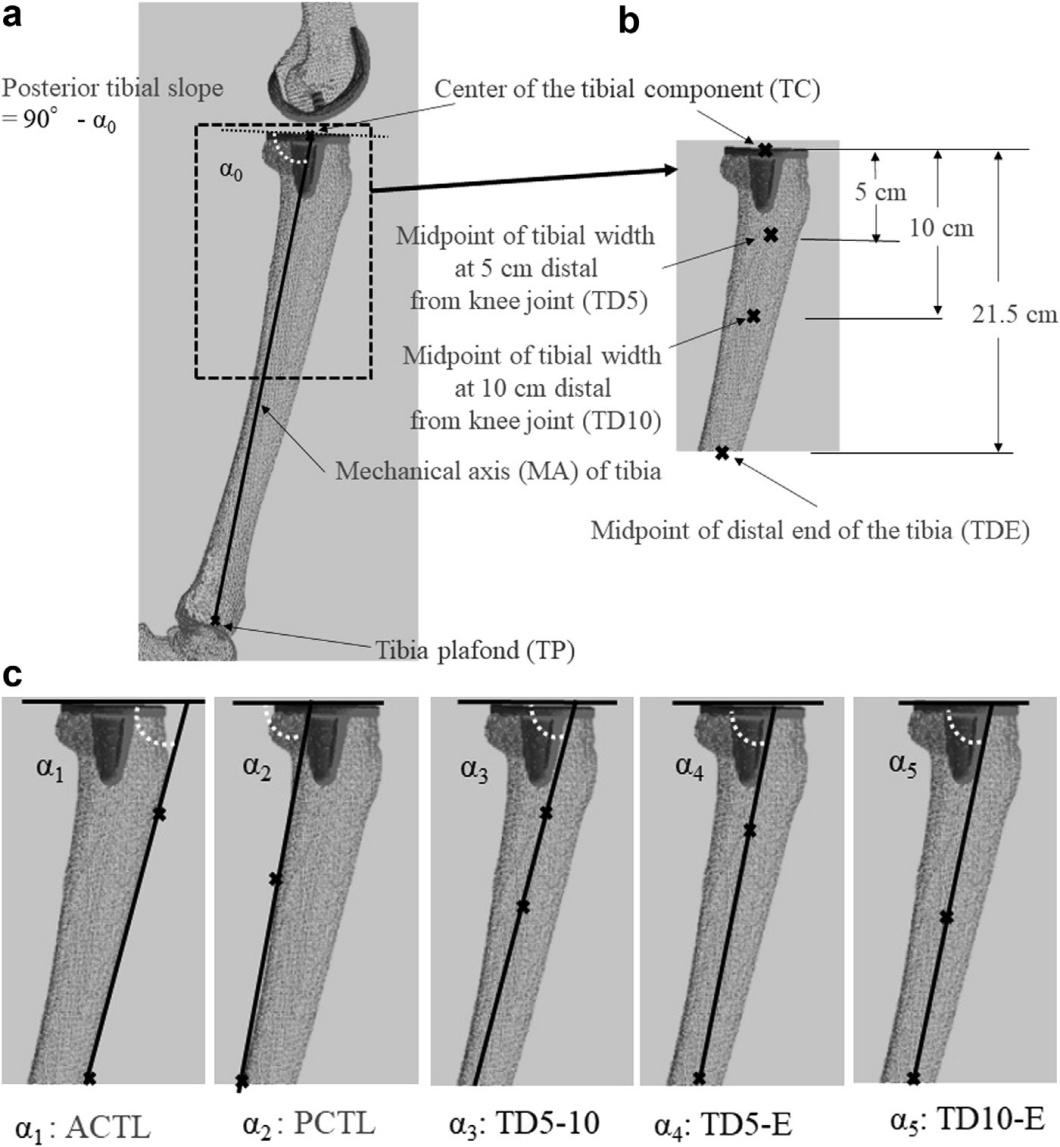
Cross-sectional views from the 3-dimensional digital model of the tibial prosthesis and bone complex in the sagittal plane are shown. The images, including the full-length lateral tibial radiograph, were used as FLTR (a), and the distal half of the lateral standard knee radiographs (36 × 43 cm) were used as LSKR (b). Posterior tibial slope was measured with reference to the sagittal MA (a), ACTL, PCTL, TD5-10, TD5-E, and TD10-E (c). ACTL, anterior cortical tibial line; PCTL, posterior cortical tibial line.

This study obtained many consistent lateral views from the 3D-constructed information using CT, which is likely to have fewer effects of tibial rotation than 2-dimensional (2D) evaluation using conventional radiographs that have limited accuracy and reproducibility for detailed investigations [[16], [17], [18], [19]]. A quantitative 3D technique developed by Sato et al. [20,21] was used. This assessment required the acquisition of preoperative CT images of each patient’s femur and tibia. Additionally, biplanar computed radiography (CR) images of the lower extremities were obtained before and after TKA. Biplanar CR images were downloaded using a 3D lower extremity alignment assessment system (Knee CAS; LEXI, Inc., Tokyo, Japan). Next, 3D digital bone and component models were projected onto biplanar CR images using the camera calibration technique. The silhouettes of these digital models were matched with the contours of the respective bone images and component CR images through 3D rotation and translation, allowing for the computation of the 3D position and alignment of the components relative to the femur and tibia. After these image-matching procedures, a 3D view of the digital model complex was displayed, in which the component models were implanted in the bone models. Any rotations between points in the 3D digital model were computed, and a cross-sectional view of the 3D digital model complex was displayed for all planes. More detailed information about this system has been published previously [[20], [21], [22], [23]]. In this study, a 3D digital model complex was selected, in which the tibial component overlapped as exactly as possible on the sagittal plane, which is regarded as a lateral view to determine the PTS (Fig. 1a–c).

The images, including the full-length lateral tibial radiograph, were used as FLTR (Fig. 1a). The proximal 21.5 cm of FLTR was used to simulate the distal half of standard knee radiographs (36 × 43 cm) as LSKR (Fig. 1b). To determine the tibial diaphyseal axes and mechanical axis, the reference points were defined from the FLTR and LSKR as mentioned below (Fig. 1a and b). The center of the tibial component is TC and that of the tibial plafond is TP. In addition, the midpoint of the distal tibial (TD) width was TD5 at 5 cm and TD10 at 10 cm from the knee joint and the end of the radiograph (TDE), respectively. Accordingly, the angle formed by the tangent line of the knee joint surface and the line connecting the points between TC and TP (mechanical axis of the tibia) was defined as α_0_, the anterior cortical tibial line was α_1_, the posterior cortical tibial line was α_2_, the line connecting TD5 and TD10 (TD5-10) was α_3_, the line connecting TD5 and TDE (TD5-E) was α_4_, and the line connecting TD10 and TDE (TD10-E) was α_5_ (Fig. 1c).

### Reproducibility

To examine the reproducibility of this method, 2 observers measured all angles (α_0_–α_5_) twice, with a 1-month interval, using a subset of 20 radiographs. Intraobserver and interobserver reliabilities were evaluated with intraclass correlation coefficients (ICCs). The ICCs for the intraobserver and interobserver reliabilities were >0.90 for all measurements (Table 2). On the basis of the reliability observed above, measurements made by a single investigator were used in the analyses.

**Table 2:** Intraclass correlation coefficients for intraobserver and interobserver reliabilities.

Variables	Intra-	Inter-
α_0_	0.944	0.941
α_1_	0.956	0.924
α_2_	0.911	0.908
α_3_	0.908	0.903
α_4_	0.971	0.962
α_5_	0.975	0.969

### Statistical analyses

Data normality was confirmed using the Q–Q plot, Kolmogorov-Smirnov test, and Shapiro-Wilk test; representative data were presented as the means and standard deviations. The correlation between 2 continuous variables was analyzed using Pearson’s correlation coefficient. The strength of the correlation of the coefficients was defined as follows: 0.70–1.00, strong; 0.40–0.69, moderate; and 0.20–0.39, weak. The Bland-Altman plot was used to determine the difference between α_0_ and α_5_, and the percentage of the number of knee joints within the tolerance limits of α_0_-α_5_ differences was calculated. The tolerance limit was set to within 2° because it is a clinically acceptable variation on radiographic measurements [24,25]. The equivalence test was performed to determine whether α_0_ and α_5_ were statistically equivalent within a difference of 2°. Intrarater and interrater reliabilities were assessed using ICCs. Statistical analyses were performed using IBM SPSS Statistics version 23 (IBM Japan, Tokyo, Japan). In all tests, *P* < .05 was considered significant.

## Results

**Figure 2:**
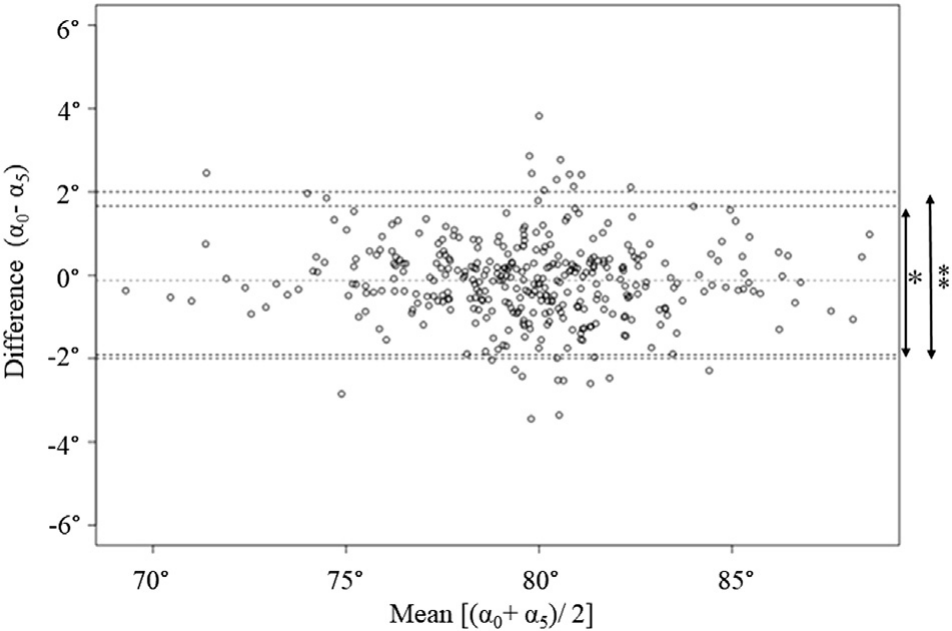
Bland-Altman plot was used to study the difference between α_0_ and α_5_. The upper and lower lines represent the 95% confidence interval of the α_0_-α_5_ differences (∗) and a clinically acceptable range of 2° (∗∗).

Each of the 5 PTS measured using LSKR showed a strong correlation with α_0_ measured using FLTR (Table 3). The weakest correlation was observed between α_3_ and α_0_ (r = 0.807, *P* < .001). The strongest correlation was observed between α_5_ and α_0_ (r = 0.948, *P* < .001). The difference between the PTS measured using LSKR and that measured using FLTR was the smallest between α_0_ and α_5_ (0.13 ± 1.00). Therefore, the possibility of using α_5_ as a substitute for α_0_ was investigated in subsequent analyses. The percentage of joints where the difference between α_0_ and α_5_ was within 2° was 93.8% of the total number of joints. The Bland-Altman plot that showed agreement between α_0_ and α_5_ is shown in Figure 2. The upper and lower lines represent the tolerance limits of the α_0_-α_5_ differences. Because a difference of ≤2° is acceptable as a measurement error for diagnostic imaging [24,25], an equivalence test was performed to determine if α_0_ and α_5_ could be considered equivalent within a tolerance limit of 2°. The equivalence test showed that α_0_ and α_5_ were statistically equivalent within the difference of 2° (*P* < .001).

**Table 3:** Results of each PTS and the comparison of PTS between α_0_ using FLTR and others using LSKR.

Number (355 knees)	Mean (SD)	Range	Difference from α_0_ (°) α_0_-α_(1-5)_	Correlation with α_0_*P* < .001^a^	Difference from α_0_ < 2° Number (%)
α_0_	79.65 (3.06)	69.11-89.05	3.69 (1.50)	.884^a^	41 (11.5)
α_1_	75.96 (3.14)	66.52-84.38			
α_2_	81.83 (3.25)	71.25-91.74	−2.18 (1.41)	.901^a^	159 (44.8)
α_3_	81.28 (3.40)	68.93-92.46	−1.63 (2.04)	.807^a^	188 (52.9)
α_4_	80.11 (3.13)	69.78-89.20	−0.46 (1.07)	.940^a^	327 (92.1)
α_5_	79.78 (3.12)	69.48-88.66	−0.13 (1.00)	.948^a^	333 (93.8)

SD, standard deviation.

a *P* < .001

## Discussion

The most important finding in this study is that PTS (α_5_) as measured on LSKR demonstrated the strongest correlation with FLTR (α_0_). In addition, within a clinically acceptable tolerance of 2°, α_0_ and α_5_ were statistically equivalent. This suggests that PTS (α_5_) can be clinically used as a substitution for PTS determined using FLTR (α_0_).

The agreement between sagittal plane alignment evaluation on LSKR vs FLTR remains unclarified, especially regarding the measurement of the PTS after TKA. Several comparative studies between them were conducted in the native knee [6], ACL-injured knee [11,12], or OA knee before TKA [13] using conventional 2D radiographs. Faschingbauer et al. [6] concluded that LSKR leads to the overestimation of PTS and provides less reproducible results. Simultaneously, they stated that only an estimation of the PTS can be performed on LSKR. Dean et al. [11] reported a significant difference between PTS measurements that used the anatomic axis in LSKR and those that used the mechanical axis of the tibia in FLTR. These studies were not consistent with the present study. Yoo et al. [13] reported that 1 (the line connecting the midpoints of the outer cortical diameter at 5 and 15 cm distal to the knee joint) of 5 anatomical axes could be drawn in an LSKR, making it possible to evaluate the sagittal alignment with radiographs obtained at routine follow-ups instead of mechanical axis in an FLTR. Their results supported the results of the present study.

Two possible factors, the different radiographic evaluation and tibial conditions between the present and previous studies, were speculated for these conflicting results. First, the evaluation was performed using cross-sectional views obtained from 3D images in the present study; unlike 2D images obtained from conventional radiographic evaluation, this study evaluated almost consistent images with minimum rotation-induced bias in the coronal and axial planes, which are the main causes of measurement errors in alignment studies [16,18,19].

Second, the conditions of both condyles of the tibia in non-TKA knees, such as native, ACL-injured, and OA knees, were different from those of TKA knees. For instance, non-TKA knees have an asymmetric size [26,27] and different posterior inclinations [[27], [28], [29]] between the medial and lateral condyles although TKA knees in this study were symmetric and had similar inclination between them. That indicates that measurement errors are likely to occur in determining the midpoint of the width of condyles of different sizes and drawing the tangent line of different inclinations between condyles in previous non-TKA knees. Because the tibial component design in this study was symmetric and both condyles were osteotomized with a posterior inclination of 10° to the mechanical axis during surgery, all measurements were performed with cross-sectional images that exactly overlapped both tibial condyles. Thus, both the midpoint of the component and tangent lines of the articular surfaces were easily determined with minimum errors in TKA knees. Such different radiographic evaluation and tibial conditions in TKA knees from those noted in previous studies with non-TKA knees may lead to conflicting results regarding the compatibility of PTS between FLTR and LSKR.

This study has several limitations. First, the study was conducted for Japanese patients only and did not consider racial differences. Shao et al. [30] reported differences in tibial shaft anatomy between Caucasians and East Asian individuals. Therefore, these findings may not apply to all patients. Second, this was a single-center study with a single prosthetic design, which limits the generalizability of the study findings. The findings of the present study should be verified through future studies conducted with various prosthetic designs and at multiple facilities. Finally, the present study analyzed CT and CR images, which are less susceptible to rotational errors than 2D plain radiographic images. However, most clinicians obtain only 2D plain radiographs prior to TKA, and it is unclear whether the present findings are generalizable to measurements on plain radiographs. Despite these limitations, the strength of this study is that it used images corresponding to 3D elements rather than conventional radiographs, which are associated with uncertainty in data accuracy and reproducibility because of 3D elements such as torsion and bowing.

## Conclusions

Considering that LSKR is more commonly used in daily clinical practice, it is crucial to determine the closest estimation of PTS using LSKR instead of that using FLTR. PTS (α_5_) based on LSKR can be used as a clinically reliable substitution of the PTS (α_0_) based on FLTR for evaluating sagittal alignment after TKA during the follow-up period.

## Conflicts of interest

The authors declare there are no conflicts of interest.

For full disclosure statements refer to https://doi.org/10.1016/j.artd.2022.06.015.

## Ethical review committee statement

The local institutional review board approved this study (the Research Board of Healthcare Corporation Ashinokai, Gyoda, Saitama, Japan [ID number: 2021-1]). All patients provided informed consent.

## Appendix A Supplementary Data

Conflict of Interest Statement for H. Ishii

Conflict of Interest Statement for Noguchi

Conflict of Interest Statement for Sato

Conflict of Interest Statement for Toyabe

Conflict of Interest Statement for K. Ishii

Conflict of Interest Statement for Takahash

Conflict of Interest Statement for Y. Ishii

Conflict of Interest Statement for R. Ishii
