# Supplementary material for: Comparison of the Tibial Posterior Slope Angle Between the Tibial Mechanical Axis and Various Diaphyseal Tibial Axes After Total Knee Arthroplasty
Source: Arthroplast Today. 2022 Sep 19;17:137–41. doi: 10.1016/j.artd.2022.06.015 (PMC9493290; doi:10.1016/j.artd.2022.06.015)
Supplement: Conflict of Interest Statement for Toyabe [file mmc4.pdf]

CONFLICT OF INTEREST STATEMENT

The Journal of Arthroplasty

(Adopted from the American Academy of Orthopaedic Surgeons disclosure statement)

The following form **must be filled out completely and submitted by each author (example, 6 authors, 6 forms). If no discloser is required, please write/type "none" at the end of each sentence.**

Comparison of the tibial posterior slope angle determined by between the tibial mechanical axis and various diaphyseal tibial axes after total knee arthroplasty

Manuscript Title

1. Royalties from a company or supplier (The following conflicts were disclosed) **none**

2. Speakers bureau/paid presentations for a company or supplier (The following conflicts were disclosed) **none**

3A. Paid employee for a company or supplier (The following conflicts were disclosed) **none**

3B. Paid consultant for a company or supplier (The following conflicts were disclosed) **none**

3C. Unpaid consultants for a company or supplier (The following conflicts were disclosed) **none**

4. Stock or stock options in a company or supplier (The following conflicts were disclosed) **none**

5. Research support from a company or supplier as a Principal Investigator (The following conflicts were disclosed) **none**

6. Other financial or material support from a company or supplier (The following conflicts were disclosed) **none**

7. Royalties, financial or material support from publishers (The following conflicts were disclosed) **none**

8. Medical/Orthopaedic publications editorial/governing board (The following conflicts were disclosed) **none**

9. Board member/committee appointments for a society (The following conflicts were disclosed) **none**

Each author must sign AND print or type his/her name, date and submit a separate form

In addition, one BLINDED Conflict of Interest form (no author names used) should be submitted per manuscript with all author disclosures.

Shin-ichi Toyabe — S Toyabe — 25/ February 2022

Author Name (Print or Type) — Author Signature — Date
